# Supplementary material for: Lifespan reference curves for harmonizing multi-site regional brain white matter metrics from diffusion MRI
Source: Sci Data. 2025 May 6;12:748. doi: 10.1038/s41597-025-05028-2 (PMC12056076; doi:10.1038/s41597-025-05028-2)
Supplement: Supplementary file 1 — Supplementary Materials [file 41597_2025_5028_MOESM1_ESM.docx]

Supplementary Information

Table of Contents

Supplementary Figure 1: ABCD Site Effects …………………………… 2

Supplementary Figure 2: LOESS parameter testing …………………………… 3

Supplementary Figure 3: Outlier Lifespan Trajectories …………………………… 4

Supplementary Figure 4: Lifespan Trajectories with ICV covariate …………………… 5

Supplementary Figure 5: Regional Shift vs Voxel Volume Plots …………………… 6

Supplementary Figure 6: Age Effects in Template vs Site …………………………… 7

Supplementary Table 1: LOESS-Derived Age Peaks …………………………… 8

Supplementary Table 2: Sex Effects …………………………………………… 9

Supplementary Table 3: ApoE4 Effects …………………………………………… 10


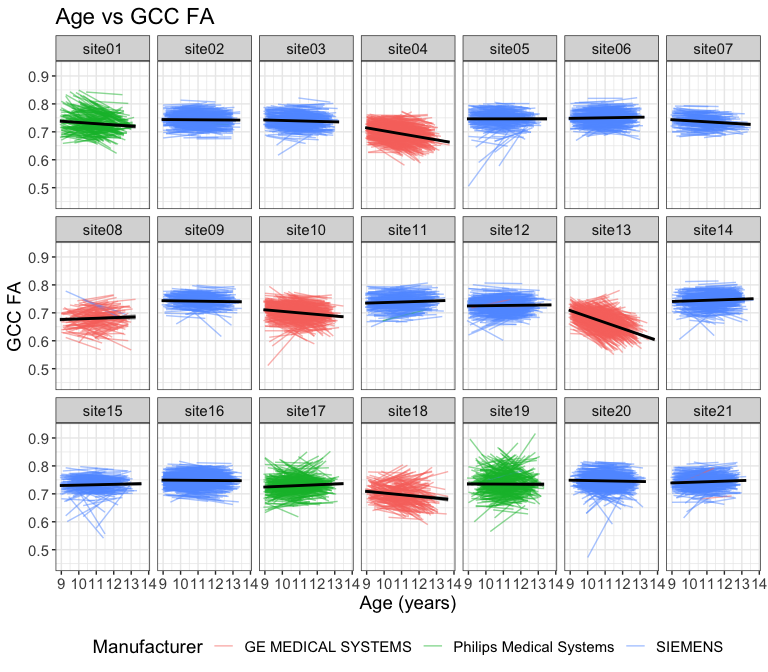


**Supplementary Figure 1.** ABCD site effects on FA in the genu of the corpus callosum (GCC). Longitudinal data from ABCD subjects are provided as spaghetti plots, separated by site and colored by manufacturer. The trend line per site is also included in black. Most sites with a GE scanner showed decreasing FA with age, contrary to the expected age effect. Philips was the least common scanner manufacturer. One Philips site had a downward trajectory (site01), and another had widely diverging subject-level trajectories (site19). As a result, we elected to use only ABCD data acquired on Siemens scanners.


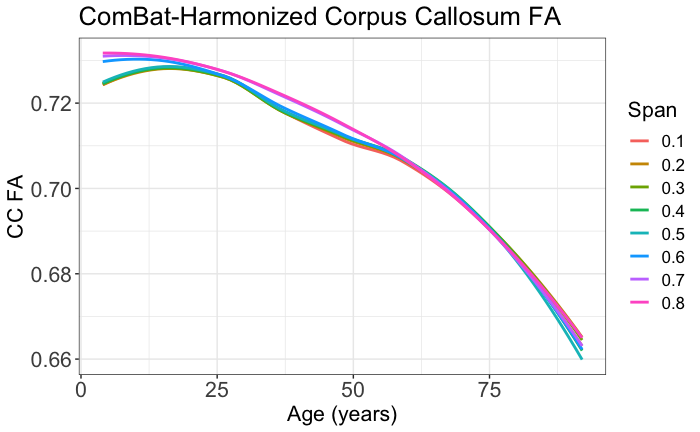
**Supplementary Figure 2.** LOESS parameter testing. We ran the LOESS model with a wide range of values for the span parameter: 0.10-0.80 by increments of 0.10 (using the R function *loess*, the default is 0.75).

**
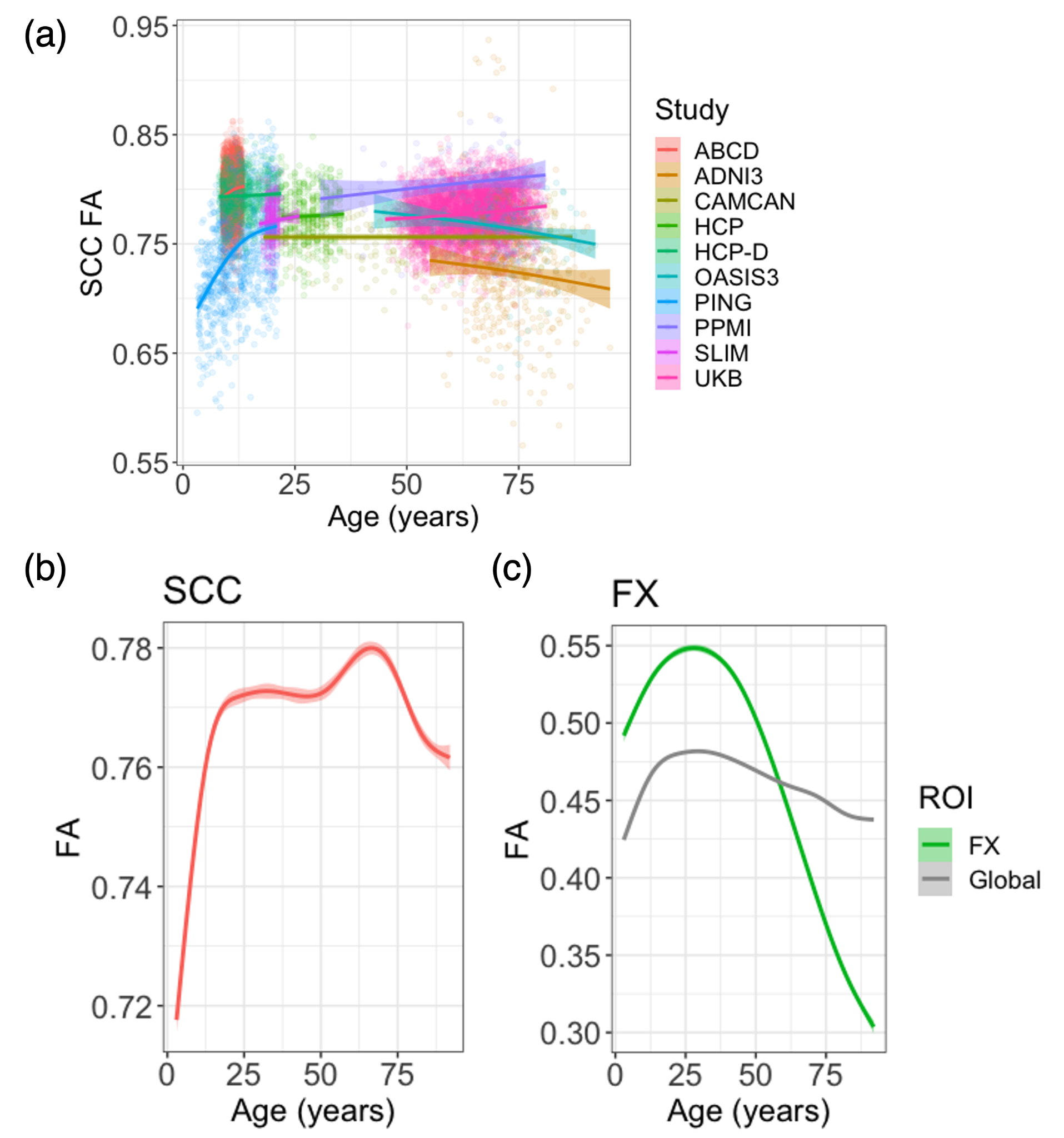
**

**Supplementary Figure 3.** Outlier ROI lifespan trajectories. FA in the SCC peaks at age 68 years, much later than all other ROIs, as can be seen in the **(a)** unharmonized data and the **(b)** modeled lifespan trajectory. **(c)** After peaking at age 29 years, FA in the FX decreases faster than in any other ROI. The lifespan reference curve for the global FA measure is also included for reference.

**
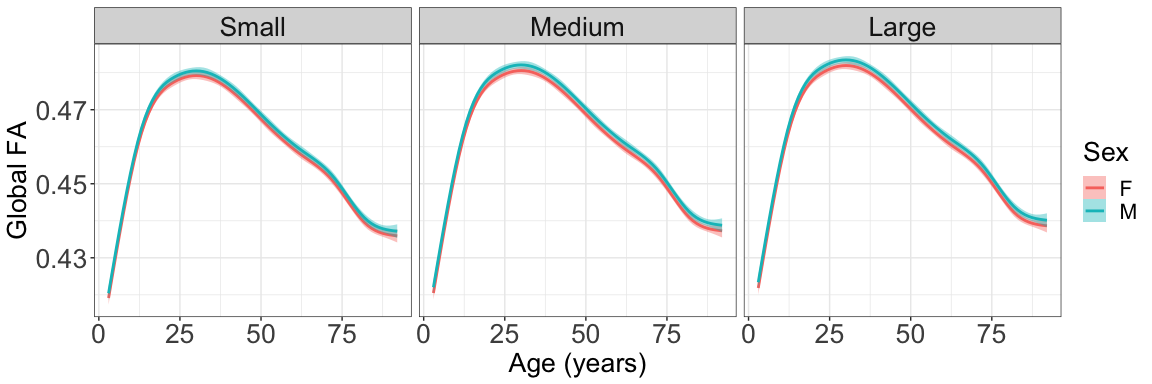
**

**Supplementary Figure 4.** When building lifespan reference curves with sex-normalized ICV as a covariate, higher FA values are observed with larger head sizes, but there is no change in the regional directionality of the sex effects.

*Female:* Small = 1300 cm^3^, Medium = 1375 cm^3^, Large = 1450 cm^3^

*Male:* Small = 1435 cm^3^, Medium = 1525 cm^3^, Large =1600 cm^3^


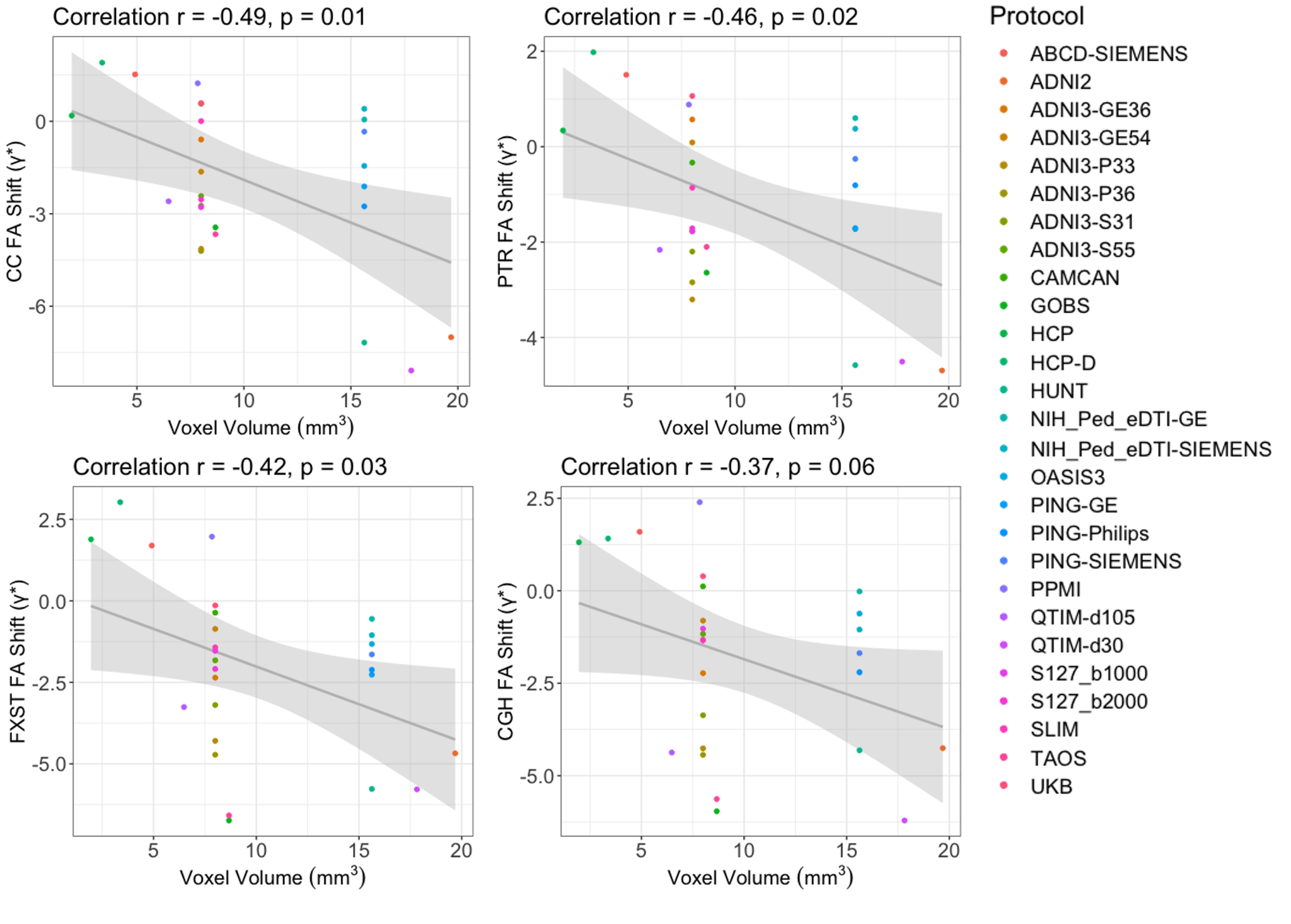
**Supplementary Figure 5.** Shift vs. voxel volume plots for the corpus callosum (CC), posterior thalamic radiation (PTR), fornix/stria terminalis (FXST), and hippocampal cingulum (CGH) display a range of negative correlations.

**
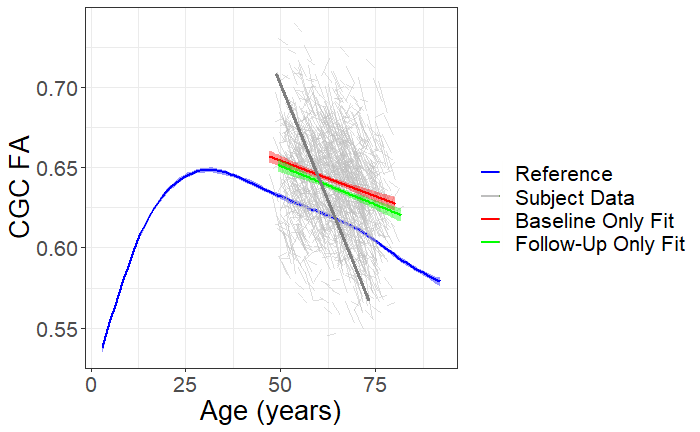
**

**Supplementary Figure 6.** In the UK Biobank, pre-harmonization cingulate gyrus (CGC) FA vs. age models fit separately by time point were found to be offset from one another. This is likely due to the larger subject-specific age slopes (spaghetti plot in gray) in comparison to the study trends (red and green). When the harmonization models were tuned independently in each time point, the follow-up data required a smaller mean shift (* = 0.6) than the baseline data ($\gamma$* = 0.7) to match the lifespan reference curve.

**Supplementary Table 1.** The LOESS age peaks showed similar patterns to those from the GAM curves. With the exception of the SCC, which was again abnormally high across all models, the age peaks from ComBat-GAM were the only ones which consistently fell within the expected 20-40 year range. In addition to the global FA measure, our MAE comparison to Kochunov et al. (2012) included eleven regions: the BCC, CC, CGC, CGH, CR, EC, GCC, IC, SFO, SLF, and SS. We excluded the SCC and the CST (reported in Kochunov et al. (2012) as N/A) from the MAE calculations due to atypical trajectories. Lebel et al. (2012) used tractography rather than the atlas-based approach used by us and Kochunov et al. (2012), so direct comparisons may be less applicable.

| **ROI** | **ComBat** | **ComBat-GAM** | **CovBat** | **Kochunov (2012)** | **Lebel (2012)** |
| --- | --- | --- | --- | --- | --- |
| AverageFA | 17 | 36 | 14 | 32 |  |
| ACR | 14 | 29 | 14 |  |  |
| ALIC | 37 | 36 | 37 |  |  |
| BCC | 14 | 36 | 14 | 32 | 35 |
| CC | 14 | 35 | 14 | 34 |  |
| CGC | 18 | 36 | 18 | 39 | 42 |
| CGH | 38 | 37 | 38 |  |  |
| CR | 14 | 29 | 14 | 28 |  |
| CST | 83 | 27 | 83 | N/A | 35 |
| EC | 15 | 35 | 15 | 26 |  |
| FX | 14 | 35 | 14 |  | 20 |
| FXST | 17 | 37 | 17 |  |  |
| GCC | 14 | 23 | 14 | 34 | 21 |
| IC | 37 | 35 | 37 | 32 |  |
| PCR | 17 | 21 | 17 |  |  |
| PLIC | 37 | 35 | 37 |  |  |
| PTR | 17 | 35 | 17 |  |  |
| RLIC | 15 | 35 | 15 |  |  |
| SCC | 66 | 66 | 66 | 30 | 25 |
| SCR | 17 | 29 | 15 |  |  |
| SFO | 18 | 37 | 40 | 39 | 28 |
| SLF | 17 | 34 | 17 | 29 |  |
| SS | 18 | 36 | 18 | 23 |  |
| TAP | 17 | 30 | 17 |  | 36 |
| UNC | 22 | 30 | 22 |  | 28 |

**Supplementary Table 2.** Sex effects across the white matter from GAM models covarying for a smoothed age term. ROIs where males had significantly higher FA are **bolded**. ROIs where females had significantly higher FA are *italicized*.

* p < 0.05; ** p < 0.001

| **ROI** | **Estimate** | **SE** | **t-value** | **p-value** | **pFDR** |
| --- | --- | --- | --- | --- | --- |
| **AverageFA** | **0.0020** | **0.00024** | **8.4** | **< 0.001**** | **< 0.001**** |
| **ACR** | **0.0018** | **0.00037** | **4.8** | **< 0.001**** | **< 0.001**** |
| **ALIC** | **0.0042** | **0.00040** | **10.5** | **< 0.001**** | **< 0.001**** |
| BCC | -0.00078 | 0.00046 | -1.7 | 0.089 | 0.092 |
| CC | 0.00014 | 0.00038 | 0.4 | 0.71 | 0.71 |
| **CGC** | **0.010** | **0.00048** | **21.7** | **< 0.001**** | **< 0.001**** |
| **CGH** | **0.0049** | **0.00066** | **7.4** | **< 0.001**** | **< 0.001**** |
| **CR** | **0.0013** | **0.00031** | **4.1** | **< 0.001**** | **< 0.001**** |
| **CST** | **0.0082** | **0.00053** | **15.5** | **< 0.001**** | **< 0.001**** |
| **EC** | **0.0030** | **0.00034** | **8.7** | **< 0.001**** | **< 0.001**** |
| *FX* | *-0.0068* | *0.00082* | *-8.3* | *< 0.001*** | *< 0.001*** |
| *FXST* | *-0.0050* | *0.00047* | *-10.5* | *< 0.001*** | *< 0.001*** |
| **GCC** | **0.0012** | **0.00048** | **2.5** | **0.011*** | **0.013*** |
| **IC** | **0.0032** | **0.00032** | **10.0** | **< 0.001**** | **< 0.001**** |
| *PCR* | *-0.00077* | *0.00037* | *-2.1* | *0.040* | *0.046* |
| **PLIC** | **0.0041** | **0.00036** | **11.5** | **< 0.001**** | **< 0.001**** |
| *PTR* | *-0.0035* | *0.00044* | *-7.9* | *< 0.001*** | *< 0.001*** |
| **RLIC** | **0.00082** | **0.00039** | **2.1** | **0.036** | **0.043** |
| SCC | 0.00076 | 0.00041 | 1.9 | 0.063 | 0.069 |
| **SCR** | **0.0014** | **0.00037** | **3.9** | **< 0.001**** | **< 0.001**** |
| **SFO** | **0.0044** | **0.00042** | **10.6** | **< 0.001**** | **< 0.001**** |
| **SLF** | **0.0030** | **0.00035** | **8.6** | **< 0.001**** | **< 0.001**** |
| *SS* | *-0.0051* | *0.00043* | *-11.9* | *< 0.001*** | *< 0.001*** |
| *TAP* | *-0.0034* | *0.00069* | *-4.9* | *< 0.001*** | *< 0.001*** |
| **UNC** | **0.0050** | **0.00060** | **8.4** | **< 0.001**** | **< 0.001**** |

**Supplementary Table 3.** ApoE4 effects across the white matter from linear regressions covarying for age, sex, age-by-sex, and age^2^ (N = 26,902). ROIs where ApoE4 was significantly associated with lower FA after multiple comparisons correction are **bolded**.

* p < 0.05; ** p < 0.001

| **ROI** | **Estimates** | **SE** | **t_values** | **p_values** | **p_FDR** |
| --- | --- | --- | --- | --- | --- |
| **AverageFA** | **-0.020** | **0.0057** | **-3.42** | **< 0.001**** | **0.0052*** |
| ACR | -0.0081 | 0.0052 | -1.56 | 0.12 | 0.25 |
| ALIC | -0.0039 | 0.0059 | -0.67 | 0.50 | 0.63 |
| BCC | -0.0084 | 0.0060 | -1.39 | 0.16 | 0.27 |
| CC | -0.014 | 0.0060 | -2.27 | 0.02* | 0.097 |
| CGC | -0.010 | 0.0058 | -1.76 | 0.08 | 0.20 |
| **CGH** | **-0.027** | **0.0059** | **-4.61** | **< 0.001**** | **< 0.001**** |
| CR | -0.0045 | 0.0056 | -0.80 | 0.42 | 0.56 |
| CST | -0.0054 | 0.0059 | -0.92 | 0.36 | 0.50 |
| EC | -0.0013 | 0.0059 | -0.22 | 0.83 | 0.90 |
| FX | -0.0063 | 0.0044 | -1.43 | 0.15 | 0.27 |
| FXST | -0.011 | 0.0056 | -1.98 | 0.048* | 0.13 |
| GCC | -0.012 | 0.0056 | -2.10 | 0.036* | 0.13 |
| IC | -0.0060 | 0.0060 | -1.01 | 0.31 | 0.46 |
| PCR | -0.0078 | 0.0060 | -1.32 | 0.19 | 0.29 |
| PLIC | 0.00043 | 0.0060 | 0.071 | 0.94 | 0.94 |
| **PTR** | **-0.022** | **0.0057** | **-3.78** | **< 0.001**** | **0.0020*** |
| RLIC | -0.012 | 0.0061 | -2.05 | 0.040* | 0.13 |
| **SCC** | **-0.016** | **0.0059** | **-2.70** | **0.0068*** | **0.043*** |
| SCR | 0.0031 | 0.0060 | 0.51 | 0.61 | 0.69 |
| SFO | 0.00081 | 0.0060 | 0.14 | 0.89 | 0.93 |
| SLF | -0.0091 | 0.0060 | -1.52 | 0.13 | 0.25 |
| SS | -0.015 | 0.0060 | -2.56 | 0.01* | 0.052 |
| TAP | -0.0096 | 0.0060 | -1.59 | 0.11 | 0.25 |
| UNC | -0.0033 | 0.0060 | -0.54 | 0.59 | 0.69 |
